# Infraclavicular Block Attenuates Tourniquet-Induced Ischemia–Reperfusion Injury and Preserves Endothelial Function Compared with General Anesthesia in Upper Extremity Surgery: A Randomized Controlled Trial

**DOI:** 10.3390/biology15141158

**Published:** 2026-07-15

**Authors:** Elif Tarım, Sezen Kumaş Solak, Rasim Onur Karaoğlu, Serdar Demirgan

**Affiliations:** Department of Anesthesiology and Reanimation, Bağcılar Training and Research Hospital, 34200 Istanbul, Türkiye; 34et22@gmail.com (E.T.); sezenkumassolak@gmail.com (S.K.S.); serdardemirgan@hotmail.com (S.D.)

**Keywords:** infraclavicular block, general anesthesia, ischemia–reperfusion, flow-mediated dilatation, oxidative stress, endothelial function, tourniquet, regional anesthesia, malondialdehyde, nitric oxide

## Abstract

**Background:** Tourniquet application during upper extremity surgery induces ischemia–reperfusion (IR) injury, generating oxidative stress and impairing endothelial function. Whether infraclavicular block (IB) or general anesthesia (GA) differentially modulates these responses remains uncertain. **Methods:** In this prospective randomized controlled trial, 124 patients were assessed for eligibility; 112 were randomized to IB (*n* = 60) or GA (*n* = 52). Following per-protocol exclusions (9 from IB, 3 from GA), 100 patients were analyzed (IB *n*= 51, GA *n* = 49). The primary outcome was change in brachial artery flow-mediated dilatation (FMD) from baseline at 24 h (FMD2) and 6 days (FMD3) postoperatively. Secondary outcomes included serum malondialdehyde (MDA), protein carbonyl (PC), total nitric oxide (NO), and xanthine oxidase (XO) measured at four perioperative time points (T0–T3). Analysis of covariance (ANCOVA) with baseline values as covariates was the primary analytical approach. **Results:** Baseline FMD was comparable between groups (IB: 6.33% vs. GA: 6.11%, *p* = 0.478). Both groups exhibited significant postoperative FMD reduction compared with the baseline; however, the magnitude of this decline was significantly smaller in the IB group than in the GA group at 24 h (ΔFMD: −1.44% vs. −1.69%, *p* = 0.004) and at 6 days (ΔFMD: −0.77% vs. −1.01%, *p* = 0.003). ANCOVA confirmed a significant independent group effect for FMD at both time points (FMD2: F = 13.95, *p* = 0.0003, η^2^p = 0.126; FMD3: F = 16.45, *p* = 0.0001, η^2^p = 0.145). Regarding oxidative stress biomarkers, baseline differences were observed in MDA and PC between groups. Following ANCOVA adjustment, no consistent group effect was identified for MDA or PC. Isolated significant group effects were noted for NO at T3 (*p* = 0.004) and XO at T1 (*p* = 0.036), but these were not sustained across time points and are considered exploratory. A sensitivity intention-to-treat analysis including all randomized patients yielded directionally consistent FMD findings, supporting the robustness of the primary results. **Conclusions:** Infraclavicular block significantly attenuated the postoperative decline in endothelial function compared with general anesthesia following tourniquet-induced IR injury, as reflected by ANCOVA-adjusted FMD outcomes with large effect sizes. The effect on oxidative stress biomarkers was inconsistent after baseline adjustment and should be considered hypothesis-generating. These findings suggest that regional anesthesia may offer endothelioprotective benefits in upper extremity surgery; however, the absence of consistent oxidative stress biomarker effects after baseline adjustment precludes conclusions regarding broader attenuation of ischemia–reperfusion injury. Confirmation in future adequately powered studies is warranted.

## Simple Summary

Tourniquet application during upper extremity surgery causes temporary interruption of blood flow followed by reperfusion, which can damage blood vessel walls. This study compared two anesthetic approaches—infraclavicular nerve block and general anesthesia—to determine whether the choice of anesthesia affects vascular injury in this setting. We found that patients who received nerve block had significantly better preservation of blood vessel function after surgery, as measured by a non-invasive ultrasound technique called flow-mediated dilatation. These findings suggest that regional anesthesia may protect blood vessels during tourniquet-based surgery, although further studies in broader patient populations are needed to confirm the clinical significance of this effect.

## 1. Introduction

Tourniquet application during upper extremity surgery provides a bloodless operative field but inevitably produces a period of tissue ischemia followed by reperfusion upon deflation [1]. This ischemia–reperfusion (IR) sequence triggers a cascade of pathophysiological events, including reactive oxygen species (ROS) generation, inflammatory pathway activation, and endothelial dysfunction [2,3]. The clinical consequences range from transient biochemical changes to more significant vascular and tissue damage, particularly in prolonged procedures [4].

Endothelial dysfunction, characterized by reduced nitric oxide bioavailability and impaired vasodilatory capacity, is a central feature of IR injury and can be reliably assessed non-invasively by brachial artery flow-mediated dilatation (FMD) [5,6]. FMD reflects endothelium-dependent, nitric oxide-mediated vasodilation and correlates with systemic vascular health and cardiovascular risk [7]. Postoperative impairment of FMD has been documented following various surgical interventions and reflects the systemic burden of oxidative and inflammatory stress [8].

Circulating oxidative stress biomarkers—including malondialdehyde (MDA), a byproduct of lipid peroxidation; protein carbonyls (PC), a measure of oxidative protein modification; total nitric oxide (NO), reflecting both vasodilatory and nitrosative activity; and xanthine oxidase (XO), a key enzymatic superoxide source during reperfusion—are widely used to quantify the extent of IR-induced cellular injury [9,10,11,12].

The anesthetic technique employed may significantly influence the perioperative stress response and, consequently, the degree of IR-induced injury. General anesthesia (GA) has been associated with varying degrees of oxidative stress modulation attributable to systemic drug effects on cellular metabolism and immune activation [13]. Regional anesthesia, in contrast, attenuates the neuroendocrine and sympathetic stress response, potentially limiting the magnitude of IR injury [14,15].

Infraclavicular brachial plexus block (IB) is a widely used regional technique for upper extremity surgery that provides reliable surgical anesthesia and prolonged postoperative analgesia while preserving systemic hemodynamic stability [16]. By blocking afferent nociceptive input and sympathetic outflow to the operative limb, IB may attenuate both local and systemic inflammatory and oxidative responses triggered by tourniquet application [17]. However, direct comparative evidence between IB and GA on endothelial function and oxidative stress markers in this clinical setting remains scarce.

Previous studies examining regional versus general anesthesia effects on perioperative oxidative stress have yielded conflicting results, often limited by small sample sizes, heterogeneous populations, and inconsistent outcome measurement [18,19]. Furthermore, FMD has rarely been incorporated as a primary endpoint in anesthesia comparison trials, despite its clinical relevance as a functional vascular marker [20].

The aim of the present randomized controlled trial was to compare the effects of infraclavicular block and general anesthesia on endothelial function as measured by FMD and on circulating oxidative stress biomarkers in patients undergoing elective upper extremity surgery under tourniquet. We hypothesized that IB would be associated with better preservation of postoperative endothelial function compared with GA.

## 2. Materials and Methods

### 2.1. Study Design and Ethics

This prospective, single-center, parallel-group, randomized controlled trial was conducted in accordance with the Declaration of Helsinki and approved by SBÜ Bakırköy Dr. Sadi Konuk Training and Research Hospital Clinical Research Ethics Committee (Decision No: 2023/10, 22 May 2023). The study was prospectively registered in a publicly accessible clinical trials database (Registration No: NCT06515028). Written informed consent was obtained from all participants prior to enrollment. This manuscript was prepared and reported in accordance with the CONSORT 2025 guidelines.

### 2.2. Participants

Adult patients aged 18–65 years with American Society of Anesthesiologists (ASA) physical status I, scheduled for elective upper extremity surgery requiring pneumatic tourniquet application, were assessed for eligibility. Exclusion criteria were: ASA physical status II or above; age outside the specified range; known peripheral vascular disease, diabetes mellitus, or arterial hypertension; active or recent smoking; obesity (BMI > 35 kg/m^2^); coagulation disorders; allergy to local anesthetics; previous brachial plexus injury; use of antioxidant or vasoactive medications; and refusal of regional anesthesia.

### 2.3. Randomization and Allocation Concealment

Eligible patients were randomized in a 1:1 ratio to IB or GA using a computer-generated random number sequence prepared by an investigator not involved in patient care. Allocation concealment was achieved using sequentially numbered, sealed, opaque envelopes opened immediately before anesthetic induction by a research nurse not involved in outcome assessment. Outcome assessors (sonographer for FMD, laboratory personnel for biomarkers) were blinded to group allocation throughout the study.

### 2.4. Anesthetic Technique

All patients received standard monitoring including electrocardiography, pulse oximetry, and non-invasive blood pressure measurement. In the IB group, ultrasound-guided infraclavicular brachial plexus block was performed with the patient in the supine position. A high-frequency linear probe was used to identify the axillary artery and surrounding cords at the infraclavicular fossa. A total of 40 mL of local anesthetic solution (20 mL 0.5% bupivacaine [Buvicaine, Polifarma İlaç San. ve Tic. A.Ş., Tekirdağ, Türkiye] combined with 20 mL 2% lidocaine [Aritmal, Osel İlaç San. ve Tic. A.Ş., İstanbul, Türkiye]; maximum dose calculated per body weight and confirmed to be within safe limits for all patients, including those of lower body weight) was administered in a perivascular distribution around the posterior cord. Intravenous midazolam (VEM İlaç San. ve Tic. A.Ş., İstanbul, Türkiye; 0.02 mg/kg) was administered for procedural sedation and anxiolysis. This sedation dose was considered unlikely to independently influence hemodynamic parameters or oxidative stress markers; however, its potential contribution cannot be entirely excluded and is acknowledged as a limitation. Patients in the IB group remained awake and cooperative throughout the surgical procedure. The midazolam dose administered was intentionally kept at the anxiolytic level (0.02 mg/kg) to avoid sedation-induced loss of consciousness, and no additional sedative agents were administered intraoperatively. Patients were verbally contactable at all times during surgery. Patients in the GA group received propofol (VEM İlaç San. ve Tic. A.Ş., İstanbul, Türkiye; 2–2.5 mg/kg) for induction, fentanyl (Fentaver, Haver Farma İlaç A.Ş., Beykoz/İstanbul, 1–2 µg/kg) for analgesia, and sevoflurane (1–2%) in a 50% oxygen/air mixture for maintenance. Neuromuscular blockade was achieved with rocuronium (Esmeron, Organon, Oss, The Netherlands; 0.6 mg/kg) and reversed with sugammadex (Sugaver, Haver Farma İlaç A.Ş., İstanbul, Türkiye) at the end of surgery. A standardized pneumatic tourniquet pressure of 250 mmHg was applied to all patients. Postoperative analgesia was standardized across both groups. All patients received paracetamol 1 g (Parol, Atabay İlaç San. ve Tic. A.Ş., İstanbul, Türkiye) intravenously every 8 h as scheduled analgesia. Rescue analgesia with tramadol (Contramal, Abdi İbrahim İlaç San. ve Tic. A.Ş., İstanbul, Türkiye) 100 mg intravenously was administered on patient request, with a minimum interval of 8 h between doses. No NSAIDs, opioid infusions, or regional catheters were used postoperatively. Total rescue analgesic consumption did not differ significantly between groups and was therefore not included as a covariate in the primary analyses.

### 2.5. Outcome Measures

The primary outcome was the change in brachial artery FMD from baseline, assessed on the contralateral (non-operative) arm to avoid direct surgical interference with the measurement vessel. FMD was measured by a trained vascular sonographer blinded to group allocation at three time points: preoperatively (FMD1, baseline), at 24 h postoperatively (FMD2), and at 6 days postoperatively (FMD3). FMD was calculated as the percentage increase in brachial artery diameter from baseline to peak diameter following 5-min forearm cuff occlusion at a standardized pressure of 250 mmHg applied distal to the antecubital fossa, in accordance with established international guidelines [21]. Measurements were conducted under standardized environmental conditions in a quiet, temperature-controlled room following at least 8 h of overnight fasting. Participants were instructed to abstain from caffeine-containing beverages, alcohol, smoking, and strenuous physical activity for at least 12 h before each assessment. All measurements were performed at approximately the same time of day to reduce circadian variation. Arterial diameter measurements were obtained manually from ultrasound images, as automated edge-detection software was not available. Intraobserver variability analysis was not formally performed, which should be considered when interpreting the FMD results.

Secondary outcomes included serum concentrations of malondialdehyde (MDA, nmol/mL), protein carbonyl (PC, ng/mL), total nitric oxide (NO, µmol/L), and xanthine oxidase (XO, ng/mL), measured at four time points: immediately before tourniquet application following establishment of the allocated anesthetic technique (T0, baseline), immediately after tourniquet deflation (T1), 30 min after deflation (T2), and 24 h postoperatively (T3). Venous blood samples were collected by a research nurse blinded to group allocation and processed under standardized laboratory conditions. All four biomarkers were quantified using commercial enzyme-linked immunosorbent assay (ELISA) kits from a single manufacturer (BT Lab, Shanghai, China) according to the manufacturer’s instructions: MDA (cat. no. E1371Hu), PC (cat. no. E1426Hu), XO (cat. no. E3495Hu), and NO (cat. no. E1510Hu). The NO assay yields a single total nitric oxide value and does not resolve nitrite and nitrate fractions separately. Hemodynamic variables—mean arterial pressure (MAP) and heart rate (HR)—were recorded at corresponding time points.

### 2.6. Patient Flow and Per-Protocol Analysis

Of 124 patients assessed for eligibility, 12 were excluded prior to randomization (ASA ≥ II: *n* = 6; exclusion criteria including diabetes or hypertension: *n* = 4; declined participation: *n* = 2). A total of 112 patients were randomized: 60 to IB and 52 to GA. Following randomization, 12 patients were excluded from analysis: in the IB group, 5 patients required conversion to general anesthesia due to block failure and were excluded to preserve anesthetic technique integrity, 3 patients had unsuccessful blood sampling precluding biomarker analysis, and 1 patient had an unsuccessful FMD measurement; in the GA group, 2 patients had their surgery cancelled or substantially modified, and 1 patient had a tourniquet time below the minimum protocol threshold. The primary analysis was therefore conducted on a per-protocol basis (IB *n* = 51, GA *n* = 49). A sensitivity intention-to-treat analysis including all randomized patients (with last-observation-carried-forward imputation for missing values) was performed and yielded directionally consistent results. The RT flow diagram is provided as Supplementary Figure S1.

### 2.7. Statistical Analysis

Sample size was estimated based on published FMD data from comparable surgical populations. Assuming a clinically meaningful between-group difference of 1.5% in FMD change, a standard deviation of 2.5%, two-sided alpha of 0.05, and 80% power, a minimum of 45 patients per group was required. Accounting for an anticipated 15% dropout rate, 60 patients per group were initially enrolled.

All analyses were performed using IBM SPSS Statistics version 26.0 (IBM Corp., Armonk, NY, USA). Normality was assessed using the Shapiro–Wilk test. As most continuous variables were non-normally distributed, between-group comparisons were performed using the Mann–Whitney U test, and within-group changes from baseline were assessed using the Wilcoxon signed-rank test. Data are expressed as median (interquartile range [IQR]).

The primary analytical method for both primary and secondary outcomes was analysis of covariance (ANCOVA), with the respective baseline values (FMD1 for endothelial outcomes; T0 for biomarker outcomes) as covariates and treatment group as the between-subjects factor. This approach adjusts for pre-existing baseline differences and isolates the independent effect of anesthetic technique on postoperative outcomes. Prior to each ANCOVA, the homogeneity of regression slopes assumption was verified by testing the interaction term between the covariate (baseline value) and treatment group. No significant interactions were identified for any outcome (all *p* > 0.05), confirming that the assumption was met and that ANCOVA was appropriate for all analyses. Effect size was estimated using partial eta-squared (η^2^p), with values of 0.01, 0.06, and 0.14 corresponding to small, medium, and large effects, respectively. Supplementary ANCOVA models additionally adjusted for operative time and intraoperative MAP to assess potential hemodynamic confounding. Given the exploratory nature of the secondary biomarker analyses, no correction for multiple comparisons was applied; these results should be interpreted accordingly. Categorical variables were compared using Fisher’s exact test. A two-sided *p*-value < 0.05 was considered statistically significant for all analyses.

## 3. Results

### 3.1. Patient Flow and Baseline Characteristics

Of 124 patients assessed for eligibility, 112 were randomized and 100 were included in the per-protocol analysis (IB *n* = 51, GA *n* = 49). Baseline demographic and clinical characteristics are presented in Table 1. The groups were well matched for age, sex, and BMI. Operative time was statistically longer in the GA group when compared to the IB group (median 95 [IQR 65–130] vs. 75 [IQR 63–95] min, *p* = 0.039), though tourniquet duration was comparable between groups (*p* = 0.121). Statistically significant baseline differences between groups were observed for MDA (*p* = 0.001) and PC (*p* = 0.017), with lower values in the IB group; the underlying cause of these imbalances could not be attributed to measured demographic or clinical variables. Additional inflammatory and metabolic parameters, including C-reactive protein, albumin, and lipid profile, were not assessed at baseline, precluding a more comprehensive characterization of pre-existing systemic differences between groups. The imbalance therefore remains unexplained; potential contributing factors, including pharmacological effects of induction agents at T0, are discussed further in the limitations section.

### 3.2. Hemodynamic Variables

Baseline MAP and HR were comparable between groups (Table 2). During the intraoperative period (T1–T3), the GA group exhibited significantly lower MAP and HR compared with the IB group (*p* < 0.001 for all comparisons), reflecting the systemic cardiovascular effects of general anesthesia. These intraoperative hemodynamic differences represent a potential physiological confounder for oxidative marker release and endothelial shear stress, and were included as covariates in supplementary ANCOVA models. By postoperative day 1 and day 6, hemodynamic parameters were similar between groups (*p* > 0.05).

### 3.3. Primary Outcome: Flow-Mediated Dilatation

Baseline FMD was comparable between groups (IB: 6.33% [5.69–7.15] vs. GA: 6.11% [5.35–6.93], *p* = 0.478). Both groups demonstrated a significant postoperative reduction in FMD at 24 h and 6 days (*p* < 0.001 for both groups, Wilcoxon signed-rank test), consistent with tourniquet-induced endothelial injury. However, the magnitude of FMD decline was significantly attenuated in the IB group compared with GA at both time points (ΔFMD2: −1.44% vs. −1.69%, *p* = 0.004; ΔFMD3: −0.77% vs. −1.01%, *p* = 0.003; Table 3).

ANCOVA with baseline FMD1 as covariate confirmed a significant independent group effect at 24 h (F = 13.95, *p* = 0.0003, η^2^p = 0.126) and at 6 days (F = 16.45, *p* = 0.0001, η^2^p = 0.145), both representing large effect sizes. Supplementary ANCOVA models additionally adjusting for operative time and intraoperative MAP confirmed that the group effect on FMD remained statistically significant at both time points (FMD2: F = 10.592, *p* = 0.0016, η^2^p = 0.100; FMD3: F = 15.426, *p* = 0.0002, η^2^p = 0.140), indicating that the between-group difference in endothelial function was not accounted for by differences in operative duration or intraoperative hemodynamic depression, and that anesthetic technique itself independently contributed to the observed FMD differences.

### 3.4. Secondary Outcomes: Oxidative Stress Biomarkers

Baseline NO and XO were comparable between groups (*p* = 0.495 and *p* = 0.975, respectively), while significant differences were observed for MDA and PC as detailed below. Oxidative stress biomarker values at all time points are presented in Table 4. As noted, baseline differences in MDA and PC between groups necessitated ANCOVA-adjusted interpretation for all biomarker outcomes.

Following ANCOVA adjustment for baseline MDA (T0), no significant group effect was identified at T1 (*p* = 0.242), T2 (*p* = 0.460), or T3 (*p* = 0.169).

For PC, ANCOVA revealed a borderline significant group effect at T1 (F = 4.06, *p* = 0.047, η^2^p = 0.040), not reproduced at T2 (*p* = 0.129) or T3 (*p* = 0.957). For NO, a significant group effect emerged at T3 (F = 8.53, *p* = 0.004, η^2^p = 0.081), with higher values in the GA group; no significant differences were observed at T1 or T2. For XO, a significant group effect was identified at T1 (F = 4.55, *p* = 0.036, η^2^p = 0.045), not sustained at subsequent time points. Given the absence of a consistent pattern across time points, these biomarker findings are considered exploratory and hypothesis-generating.

## 4. Discussion

The principal finding of this randomized controlled trial is that infraclavicular brachial plexus block significantly attenuated the postoperative decline in endothelial function, as measured by brachial artery FMD, compared with general anesthesia in patients undergoing upper extremity surgery under tourniquet. ANCOVA with baseline FMD as covariate confirmed a robust, independent, and large-effect-size group difference at both 24 h and 6 days postoperatively (η^2^p = 0.126 and 0.145, respectively). These findings provide meaningful evidence that anesthetic technique influences perioperative endothelial function in the context of tourniquet-induced IR injury.

Tourniquet-induced IR injury is a recognized cause of perioperative endothelial activation and dysfunction. The abrupt cessation of limb perfusion followed by sudden reperfusion generates a burst of reactive oxygen species, particularly superoxide, which rapidly inactivates endothelium-derived nitric oxide and impairs vasodilatory function [2,3]. FMD is a validated functional surrogate of this process, capturing endothelium-dependent nitric oxide-mediated vasodilation, and has been shown to decline in experimental and clinical IR paradigms [5,6]. In the present study, both groups exhibited significant FMD reduction postoperatively, consistent with published data on tourniquet-induced endothelial injury [8]. The significantly smaller FMD decline observed in the IB group indicates that regional anesthesia conferred a measurable degree of endothelioprotection. Notably, FMD was assessed in the contralateral, non-operative arm, yet a measurable postoperative decline was detected in both groups. This indicates that a localized, tourniquet-confined ischemia–reperfusion insult was sufficient to produce endothelial dysfunction in a remote systemic vascular bed, rather than a purely regional vascular effect. This systemic propagation is consistent with the concept of remote endothelial injury, whereby circulating oxidative and inflammatory mediators released upon reperfusion impair endothelium-dependent vasodilation beyond the ischemic territory. The attenuation of this remote endothelial response in the IB group therefore suggests that regional anesthesia may modulate the systemic, and not merely the local, vascular consequences of tourniquet-induced ischemia–reperfusion. The clinical significance of these findings extends beyond the immediate perioperative period. Postoperative endothelial dysfunction, as reflected by impaired FMD, has been independently associated with increased long-term cardiovascular risk, including accelerated atherosclerosis progression and adverse cardiac events in longitudinal population studies [7]. The sustained FMD difference observed at 6 days postoperatively in the present study suggests that patients undergoing general anesthesia for tourniquet-based upper extremity surgery may experience a more prolonged period of endothelial vulnerability compared with those receiving infraclavicular block. However, the present study was not designed to evaluate clinical endpoints, and the absolute magnitude of the FMD difference was modest. Any translational cardiovascular implication therefore remains speculative and hypothesis-generating, and would need to be tested directly in trials powered for patient-centered outcomes, particularly in patients with pre-existing endothelial dysfunction or elevated cardiovascular risk.

The mechanisms underlying IB-mediated preservation of endothelial function are likely multifactorial and, in the absence of direct mechanistic measurements, remain hypothetical. First, IB blocks sympathetic outflow to the operative limb, potentially modulating local vascular tone and microvascular perfusion dynamics during the ischemic phase [17]. Second, regional anesthesia attenuates the systemic neuroendocrine stress response, reducing catecholamine and cortisol release, which are known to impair endothelial nitric oxide synthase activity [14,15]. Third, local anesthetic agents such as bupivacaine and lidocaine possess direct anti-inflammatory and membrane-stabilizing properties that may limit cellular injury during reperfusion [22,23]. Fourth, the superior hemodynamic stability observed in the IB group—reflected by significantly higher intraoperative MAP and HR compared with the GA group—may have provided more favorable conditions for endothelial recovery, as adequate perfusion pressure supports normal endothelial function [24]. The significantly longer operative time observed in the GA group (median 95 vs. 75 min, *p* = 0.039) represents a potential confounding variable, as prolonged surgery may independently exacerbate tourniquet-induced ischemic burden and systemic inflammatory responses. The reason for this between-group difference cannot be determined from the present data, and it could theoretically contribute to greater endothelial injury in the GA group independently of anesthetic effects. Importantly, supplementary ANCOVA models additionally adjusting for both operative time and intraoperative MAP did not eliminate the FMD group difference (Section 3.3), indicating that this confounding did not account for the observed endothelial benefit of IB.

The interpretation of oxidative stress biomarker results requires particular caution. Following ANCOVA adjustment, no significant group effect was identified for MDA at any time point, indicating the absence of a differential anesthetic effect on lipid peroxidation after accounting for baseline differences. A similar pattern was observed for PC, with only a single marginal ANCOVA-significant time point. These findings highlight the critical importance of baseline adjustment in the interpretation of oxidative stress biomarker data in clinical trials where random baseline imbalances occur despite randomization [25]. The divergence between robust FMD findings and largely non-significant adjusted biomarker results merits further consideration. FMD reflects integrated endothelial nitric oxide bioavailability and vasomotor function, capturing cumulative endothelial injury over hours to days. Circulating biomarkers such as MDA and PC, in contrast, represent transient snapshots of oxidative flux at discrete time points, subject to high biological variability, rapid metabolic clearance, and pre-analytical factors. It is therefore plausible that IB exerts a sustained modulatory effect on endothelial function via mechanisms—including sympathetic blockade, neuroendocrine attenuation, and local anesthetic anti-inflammatory properties—that are not reliably captured by point-in-time oxidative stress assays. Alternatively, the biomarker null findings may reflect insufficient statistical power for these exploratory secondary outcomes, particularly given the substantial baseline imbalances in MDA and PC that reduced ANCOVA sensitivity.

The elevated total NO observed in the GA group at T3 in ANCOVA warrants careful biological interpretation. Circulating NO reflects the combined output of endothelial nitric oxide synthase—a vasodilatory, cardioprotective enzyme—and inducible nitric oxide synthase, which is upregulated in the context of inflammation and tissue injury [11]. Elevated total NO in the GA group at T3 may therefore reflect ongoing nitrosative stress or compensatory upregulation of nitric oxide production in response to greater endothelial injury, rather than a direct anesthetic effect. Because our assay reports a single total NO value without resolving nitrite and nitrate, and in the absence of isoform-specific assays or direct measurement of peroxynitrite or other reactive nitrogen species, definitive mechanistic conclusions cannot be drawn. Similarly, the transient XO difference at T1 in ANCOVA, while biologically plausible given XO’s role as an early reperfusion superoxide source [12], was not sustained and should be interpreted with caution.

Our findings are broadly consistent with, and extend, prior work examining regional versus general anesthesia in the context of perioperative vascular biology. Several studies have reported attenuated inflammatory and oxidative markers with regional techniques [18,19], though direct comparisons are hampered by heterogeneous designs. Our study contributes by employing FMD—a functional and clinically validated vascular endpoint—as the primary outcome, demonstrating that the endothelioprotective effect of IB is both statistically robust and of large effect size.

Several limitations should be acknowledged. First, and most importantly, this trial enrolled exclusively ASA I patients—a highly selected, physiologically homogeneous population with no comorbidities, free from diabetes, hypertension, peripheral vascular disease, obesity, or smoking. While this design choice was deliberate to minimize confounding and isolate the effect of anesthetic technique, it substantially limits the generalizability of the findings to routine clinical practice, where the majority of surgical patients carry one or more of these risk factors. The endothelioprotective effect of infraclavicular block, if it indeed operates via neuroendocrine and sympatholytic mechanisms, may behave differently—or even more prominently—in patients with pre-existing endothelial dysfunction, in whom baseline vascular reserve is already compromised. Future studies should specifically target higher-risk populations to establish whether these findings are clinically translatable. Second, baseline differences in MDA and PC between groups, despite randomization, represent an inherent limitation. These imbalances could not be attributed to measured demographic or clinical variables and likely reflect unmeasured biological variability. The absence of baseline measurements of C-reactive protein, albumin, or lipid profile means that systematic pre-existing differences in inflammatory or metabolic status between groups cannot be excluded. This unexplained imbalance reduces the unambiguity of conclusions drawn from biomarker outcomes, even after ANCOVA adjustment. Furthermore, in the GA group, T0 samples were obtained after induction with propofol, fentanyl, and rocuronium. The sympatholytic properties of propofol, the vascular smooth muscle relaxant effects of fentanyl, and neuromuscular blockade with rocuronium may have independently influenced baseline MDA and PC values, potentially contributing to the observed between-group imbalance at T0 and representing an additional source of asymmetry that cannot be fully disentangled from pre-existing biological differences. Third, intraoperative hemodynamic differences between groups—a known consequence of comparing regional and general anesthesia—constitute a potential physiological confounder. Although supplementary adjusted models support the robustness of the primary FMD finding, residual hemodynamic confounding for biomarker outcomes cannot be excluded. Fourth, the exclusion of five patients with failed infraclavicular block from the per-protocol analysis warrants particular attention. Block failure represents a clinically meaningful event—patients in whom IB did not achieve adequate anesthesia may represent a subgroup with anatomical or physiological characteristics associated with differential outcomes, and their exclusion may artificially improve the apparent effectiveness of the IB group. The sensitivity ITT analysis, which retained all randomized patients using last-observation-carried-forward imputation, yielded directionally consistent FMD results, supporting the robustness of the primary findings. However, last-observation-carried-forward imputation may not accurately reflect the true dynamic trajectory of FMD and oxidative stress biomarkers, potentially underestimating the magnitude of postoperative changes in excluded patients. These considerations should be borne in mind when interpreting the primary per-protocol results. Fifth, the absence of clinical outcome data—including wound healing, thromboembolic events, and neurologic outcomes—means that the clinical significance of the observed endothelial differences remains to be established. The present findings should therefore be considered hypothesis-generating regarding long-term vascular outcomes. Sixth, intravenous midazolam (0.02 mg/kg) was administered exclusively to the IB group, representing a pharmacological asymmetry between groups. Midazolam has been reported to exert antioxidant effects and to induce endothelium-dependent vasodilation in experimental models [26,27,28], raising the possibility that it may have partially confounded both the biomarker and FMD comparisons. However, the low dose employed makes a substantial systemic effect unlikely. Future studies should nonetheless standardize sedation across groups or omit it entirely to eliminate this potential bias. Seventh, serum NO was quantified using an antibody-based ELISA that reports a single total value; nitrite and nitrate fractions were not resolved. Consequently, the relative contributions of endothelial and inducible nitric oxide synthase activity cannot be inferred from our data, and chemiluminescence or Griess-based nitrate-reduction methods would be required for finer characterization. Eighth, endothelin-1—the principal endothelium-derived vasoconstrictor and the counterpart to NO in the regulation of vascular tone—was not measured. The biomarker panel was defined a priori in the study protocol and did not include ET-1; simultaneous ET-1 and NO measurement would permit direct assessment of the ET-1/NO balance that underlies endothelial dysfunction, and should be incorporated into future trials. Finally, the biomarker panel employed in this study—while widely used in perioperative oxidative stress research—provides a limited mechanistic characterization of ischemia–reperfusion injury. Contemporary investigation of endothelial dysfunction and reperfusion biology increasingly incorporates inflammatory mediators (interleukin-6, tumor necrosis factor-α), endothelial activation markers (E-selectin, ICAM-1), and molecular indices of nitrosative stress. The absence of such markers limits the mechanistic depth of the present findings, which should therefore be regarded as hypothesis-generating rather than explanatory. Future studies incorporating a more comprehensive mechanistic panel would be needed to elucidate the biological pathways underlying the observed endothelial differences.

## 5. Conclusions

In patients undergoing elective upper extremity surgery under tourniquet, infraclavicular brachial plexus block significantly attenuated the postoperative decline in brachial artery flow-mediated dilatation compared with general anesthesia, with large effect sizes confirmed by ANCOVA at both 24 h and 6 days postoperatively. This finding suggests that infraclavicular block may attenuate the postoperative decline in endothelial function in the setting of tourniquet-induced ischemia–reperfusion injury; whether this confers a clinically meaningful benefit remains to be established. The effect of anesthetic technique on oxidative stress biomarkers was inconsistent following ANCOVA adjustment for baseline differences, and these results should be interpreted as exploratory and hypothesis-generating. Future multicenter trials incorporating broader patient populations, comprehensive mechanistic biomarker panels including inflammatory and endothelial activation markers, and hard clinical outcome measures are needed to establish the translational significance of these findings and to determine whether the observed endothelial differences confer meaningful clinical benefit.

## Figures and Tables

**Table 1:** Baseline demographic and clinical characteristics.

Variable	IB (*n* = 51)	GA (*n* = 49)	*p*-Value
Age (years)	39 (25–49)	39 (27–54)	0.825
Sex, Female/Male, n	21/30	13/36	0.143
BMI (kg/m^2^)	26.2 (23.4–28.4)	26.7 (24.6–28.2)	0.652
Operative time (min)	75 (63–95)	95 (65–130)	0.039 *
Tourniquet duration (min)	58 (49–77)	77 (42–108)	0.121

Data are expressed as median (interquartile range) or number. * *p* < 0.05; IB: infraclavicular block; GA: general anesthesia; BMI: body mass index.

**Table 2:** Intraoperative and postoperative hemodynamic variables.

Variable	IB (*n* = 51)	GA (*n* = 49)	*p*-Value	Time Point
MAP T0 (mmHg)	93 (87–99)	92 (84–97)	0.354	Baseline
MAP T1 (mmHg)	91 (84–95)	78 (70–84)	<0.001 ***	Intraop
MAP T2 (mmHg)	90 (83–94)	76 (69–81)	<0.001 ***	Intraop
MAP T3 (mmHg)	89 (85–95)	81 (75–89)	<0.001 ***	Intraop
HR T0 (bpm)	82 (74–85)	76 (72–83)	0.177	Baseline
HR T1 (bpm)	74 (70–78)	62 (57–65)	<0.001 ***	Intraop
HR T2 (bpm)	74 (68–78)	62 (57–65)	<0.001 ***	Intraop
HR T3 (bpm)	73 (69–77)	65 (62–70)	<0.001 ***	Intraop

Data are expressed as median (interquartile range). *** *p* < 0.001. MAP: mean arterial pressure; HR: heart rate; IB: infraclavicular block; GA: general anesthesia.

**Table 3:** Flow-mediated dilatation (FMD) values and between-group comparisons.

	IB (*n* = 51)	GA (*n* = 49)	MWU p	ANCOVA *p* (η^2^p)
FMD1, baseline (%)	6.33 (5.69–7.15)	6.11 (5.35–6.93)	0.478	—
FMD2, 24 h (%)	5.05 (3.94–6.05)	4.55 (3.21–5.45)	0.202	—
FMD3, day 6 (%)	5.56 (4.52–6.69)	5.08 (4.04–6.06)	0.238	—
ΔFMD2 − FMD1 (%)	−1.44 (−1.77 to −1.17)	−1.69 (−2.07 to −1.37)	0.004 **	0.0003 (0.126)
ΔFMD3 − FMD1 (%)	−0.77 (−1.13 to −0.58)	−1.01 (−1.34 to −0.82)	0.003 **	0.0001 (0.145)

ΔFMD2: change in FMD from baseline to 24 h postoperatively (FMD2 − FMD1). ΔFMD3: change in FMD from baseline to 6 days postoperatively (FMD3 − FMD1). Data are expressed as median (interquartile range). ANCOVA: analysis of covariance with baseline FMD1 as covariate. MWU: Mann–Whitney U test. η^2^p: partial eta-squared. ** *p* < 0.01.

**Table 4:** Oxidative stress biomarkers at all perioperative time points.

	IB (*n* = 51)	GA (*n* = 49)	ANCOVA *p* (η^2^p)
MDA (nmol/mL)			
T0 (baseline)	14.03 (10.12–17.84)	18.82 (14.14–23.26)	(baseline covariate)
T1	15.28 (10.85–21.45)	19.79 (17.37–24.46)	0.242 (0.015)
T2	16.03 (12.10–23.33)	22.39 (18.15–28.25)	0.460 (0.006)
T3	12.02 (9.04–20.12)	19.93 (16.51–23.73)	0.169 (0.026)
PC (ng/mL)			
T0 (baseline)	33.90 (22.67–86.17)	119.32 (22.12–239.45)	(baseline covariate)
T1	88.17 (22.36–153.38)	114.99 (30.91–267.58)	0.047 * (0.040)
T2	56.39 (22.59–131.16)	116.38 (39.27–233.98)	0.129 (0.024)
T3	44.38 (18.60–129.66)	137.25 (41.12–269.33)	0.957 (0.000)
NO (µmol/L)			
T0 (baseline)	216.91 (167.91–263.40)	204.81 (65.65–468.97)	(baseline covariate)
T1	238.13 (195.16–351.46)	294.59 (107.10–469.90)	0.386 (0.008)
T2	247.33 (194.59–328.13)	276.95 (123.32–398.79)	0.672 (0.002)
T3	199.21 (174.81–270.69)	283.94 (179.29–491.60)	0.004 ** (0.081)
XO (ng/mL)			
T0 (baseline)	27.31 (20.48–35.33)	27.43 (20.34–35.76)	(baseline covariate)
T1	26.82 (18.68–33.58)	26.74 (22.88–37.02)	0.036 * (0.045)
T2	22.05 (17.44–33.66)	28.45 (21.65–38.61)	0.234 (0.015)
T3	28.39 (21.33–36.13)	27.62 (20.67–42.04)	0.309 (0.011)

Data are expressed as median (interquartile range). ANCOVA: analysis of covariance with T0 as covariate. η^2^p: partial eta-squared. * *p* < 0.05; ** *p* < 0.01. MDA: malondialdehyde; PC: protein carbonyl; NO: total nitric oxide; XO: xanthine oxidase.

## Data Availability

The datasets generated and/or analyzed during the current study are available from the corresponding author on reasonable request.

## Supplementary Materials

The following supporting information can be downloaded at: https://www.mdpi.com/article/10.3390/biology15141158/s1, Figure S1: CONSORT Flow Diagram. Flow diagram showing the flow of participants through the study, including assessment for eligibility, exclusions before randomization with reasons, randomization to the infraclavicular block (IB) and general anesthesia (GA) groups, per-protocol exclusions with reasons, and the number of patients analyzed in each group.

## Abbreviations

The following abbreviations are used in this manuscript:

ANCOVA	analysis of covariance
ASA	American Society of Anesthesiologists
BMI	body mass index
FMD	flow-mediated dilatation
GA	general anesthesia
HR	heart rate
IB	infraclavicular block
IR	ischemia–reperfusion
ITT	intention-to-treat
MAP	mean arterial pressure
MDA	malondialdehyde
NO	total nitric oxide
PC	protein carbonyl
ROS	reactive oxygen species
XO	xanthine oxidase
η^2^p	partial eta-squared
