# Supplementary material for: Infraclavicular Block Attenuates Tourniquet-Induced Ischemia–Reperfusion Injury and Preserves Endothelial Function Compared with General Anesthesia in Upper Extremity Surgery: A Randomized Controlled Trial
Source: Biology (Basel). 2026 Jul 15;15(14):1158. doi: 10.3390/biology15141158 (PMC13404593; doi:10.3390/biology15141158)
Supplement: Supplementary file 1 [file biology-15-01158-s001.zip › biology-4371439-supplementary.pdf]

Supplementary Figure S1. CONSORT Flow Diagram

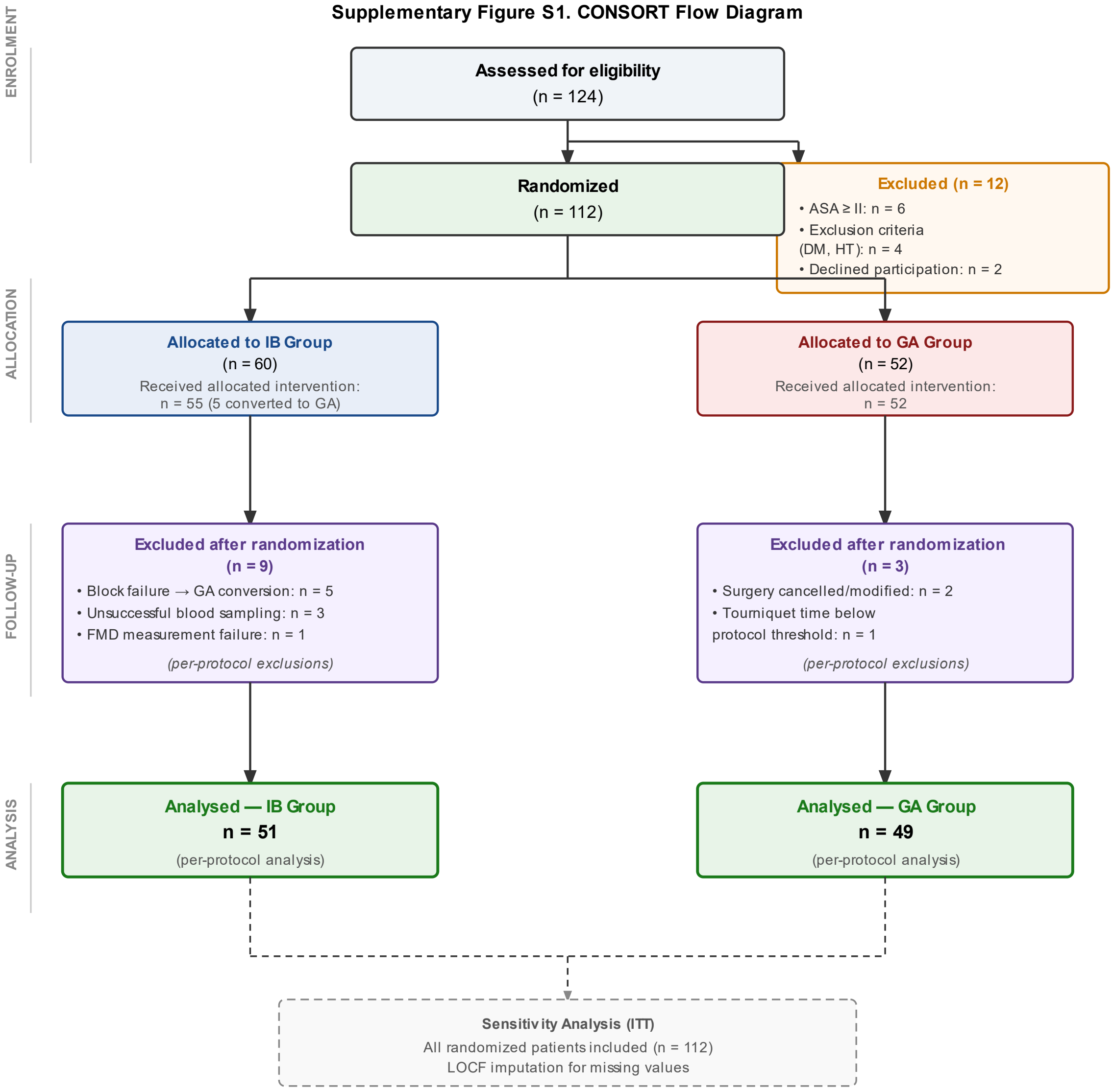

IB: infraclavicular block; GA: general anesthesia; DM: diabetes mellitus; HT: hypertension; FMD: flow-mediated dilatation; ITT: intention-to-treat; LOCF: last-observation-carried-forward.

Prepared in accordance with CONSORT 2025 guidelines.
